# Cholesterol-Driven Optimization of Liposomal Systems for Ivermectin Capture: Insights from Experimental and Molecular Dynamics Studies

**DOI:** 10.1021/acsami.5c21365

**Published:** 2026-02-16

**Authors:** Alexandre C. M. Barros, Jader Pires, Karinna Mendanha, Lucas R. de Sousa, Bianca B. Fontanezi, Guilherme Colherinhas, Ana F. M. Botelho, Sebastião A. Mendanha, Eliana M. Lima

**Affiliations:** a Laboratory of Veterinary Toxicology − School of Veterinary and Animal Sciences, Federal University of Goiás, Goiânia, Goiás 74690-900, Brazil; b FarmaTec - Laboratory for RD&I in Pharmaceutical Nanotechnology and Drug Delivery Systems, Samambaia Technology Park, UFG, Goiânia, Goiás 74690-631, Brazil; c School of Pharmacy, Federal University of Goiás, Goiânia 74690-631, Brazil; d Institute of Physics, Federal University of Goiás, Goiânia, Goiás 74690-900, Brazil

**Keywords:** lipid membrane dynamics, drug−membrane affinity, nanocarrier design, computational modeling, sterol content variation

## Abstract

This study investigates the interactions between ivermectin (IVM) and lipid membranes with varying cholesterol contents by using a combined molecular dynamics (MD) and experimental approach. DOPC bilayers containing 0, 10, 20, or 30% cholesterol were simulated, and SPC liposomes were employed for experimental validation. Mass density profiles indicated that the membrane thickness increased from 4.16 nm (0% cholesterol) to 4.60 nm (30% cholesterol), while ivermectin was most deeply embedded in membranes with 10% cholesterol with an average distance of 1.09 nm from the bilayer center. van der Waals interaction energies were most favorable at 10% cholesterol (−333.13 kJ/mol), correlating with an increased hydrogen-bond lifetime (2.10 ns) between IVM and lipid molecules. Mean square displacement (MSD) analysis revealed that ivermectin exhibited the lowest mobility (0.0019 × 10^–5^ cm^2^/s) in membranes with 10% cholesterol. ESR spectroscopy of 5-DSA-labeled SPC liposomes demonstrated a progressive increase in 2A_||_ values with increasing cholesterol content, with additional increases following IVM incorporation. IVM capture experiments showed that liposomes containing 10% cholesterol achieved the highest drug association, consistent across saline and plasma environments. These findings provide a mechanistic basis for the rational design of liposomal systems with high ivermectin-binding capacity, with potential implications for future applications requiring the sequestration of this compound in biological environments.

## Graphical abstract

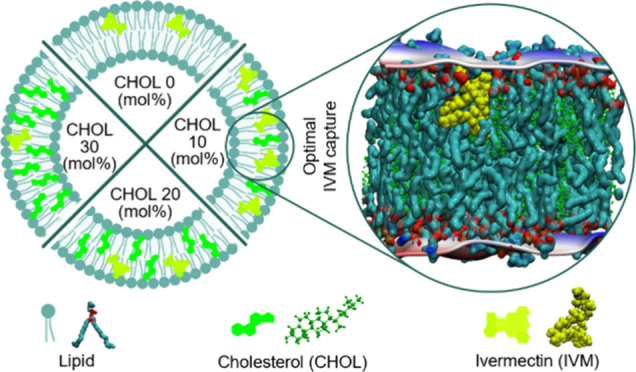

## 1 Introduction

Ivermectin, a macrocyclic lactone derived from *Streptomyces avermitilis*,^1^ is extensively used in both human and veterinary medicine owing to its strong antiparasitic and anti-inflammatory properties.^2^ From a physicochemical perspective, it exhibits high lipophilicity (log*P* ≈ 4.3–5.8) and extremely low aqueous solubility, characteristics that critically impact its pharmacokinetic behavior and interactions with biological membranes.^3,4^ Following oral administration, ivermectin is rapidly absorbed and metabolized in the liver, primarily via cytochrome P450 enzymes, reaching peak plasma concentrations approximately 4 h postdose and demonstrating a plasma half-life of around 24 h.^5,6^ Its metabolites, however, attain maximal plasma levels near 7 h and exhibit a prolonged half-life of roughly 72 h,^7^ indicating sustained systemic exposure and extended pharmacological activity. These pharmacokinetic features, combined with ivermectin’s high tissue accumulation and protein binding, raise concerns about its systemic distribution and potential toxicity, creating a strong rationale for exploring lipid-based systems capable of modulating its bioavailability through selective sequestration.

Drug–membrane interactions are central to the pharmacodynamics of bioactive compounds, particularly for hydrophobic molecules, such as ivermectin. The ability of such compounds to partition into lipid bilayers not only governs their cellular uptake and intracellular distribution but also modulates membrane properties, including fluidity, potential, and permeability, which are critical for various biological responses.^8^ Ivermectin has been shown to integrate into biological membranes, leading to increased rigidity, alterations in membrane potential, and changes in barrier function.^9^ These effects have been observed across diverse biological systems, from parasitic membranes, where ivermectin localizes preferentially to the outer leaflet of Ascaris suum muscle vesicles,^10^ to mammalian cells, where it induces membrane hyperpolarization in cell lines, such as OCI-AML2, U937, and TEX,^11^ and promotes membrane disruption in A549 pulmonary epithelial cells.^12^

Although accumulating evidence supports ivermectin’s capacity to interact with lipid membranes and alter their biophysical properties, the precise molecular determinants governing these interactions remain insufficiently characterized, especially in membranes enriched with cholesterol. Given cholesterol’s profound influence on bilayer architecture, fluidity, and permeability,^8^ understanding how its varying concentrations affect ivermectin’s partitioning, insertion depth, and lateral mobility is essential yet remains largely unexplored. An integrated approach combining molecular dynamics simulations with experimental liposome models offers a unique opportunity to dissect the molecular determinants of ivermectin–membrane interactions while validating predictions under biologically relevant conditions

Despite increasing evidence that ivermectin strongly partitions into lipid bilayers and alters their physical properties, the specific role of cholesterol in modulating these interactions is poorly understood. Cholesterol is a key regulator of membrane organization, fluidity, and permeability, and its concentration can drastically alter the behavior of embedded molecules. However, to date, no systematic study has addressed how incremental cholesterol enrichment influences the partitioning depth, stability, and capture efficiency of ivermectin within model membranes. Clarifying this relationship is essential not only for advancing the fundamental understanding of ivermectin–lipid interactions but also for guiding the rational design of liposomal systems tailored for the sequestration or delivery of hydrophobic drugs in biologically relevant environments.

To address this gap, we investigated the modulatory role of cholesterol in the molecular interactions between ivermectin and lipid bilayers. Classical molecular dynamics simulations were employed to characterize ivermectin’s spatial distribution, diffusivity, and interaction energetics in DOPC membranes containing 0, 10, 20, and 30% cholesterol. These computational insights were complemented by experimental validation using SPC-based liposomes, analyzed through electron spin resonance (ESR) spectroscopy and quantitative assessment of ivermectin incorporation. By integrating theoretical and experimental methodologies, this study aims to provide a detailed mechanistic understanding of cholesterol-dependent drug–membrane interactions. Furthermore, by identifying membrane compositions that optimize ivermectin embedding and retention, this work offers a rational framework for the design of lipid-based systems tailored for applications where efficient partitioning and sequestration of hydrophobic drugs are desired.

## 2 Materials and Methods

### 2.1 Chemicals

Soy phosphatidylcholine (SPC) Lipoid S100 was obtained from Lipoid GmbH (Germany). 1,2-Distearoyl-*sn*-glycero-3-phosphoethanolamine-poly­(ethylene glycol) 2000 (DSPE-PEG-2000), cholesterol (CHOL), ivermectin (IVM), and the spin label 5-doxyl-stearic acid (5-DSA) were purchased from Sigma-Aldrich (USA). Methanol and acetonitrile were sourced from Merck (Germany). All other chemicals and reagents used were of analytical grade or higher.

### 2.2 Molecular Dynamics Simulations

Initially, the structure of the IVM molecule was positioned on a lipid membrane containing 128 molecules of 1,2-dioleoyl-*sn*-glycero-3-phosphocholine (DOPC), formed by a bilayer of 64 molecules positioned in the *XY* plane. The simulation box was filled with water molecules, with a thickness of 6 nm. After a classical molecular dynamics (MD) simulation, conducted to achieve thermodynamic equilibrium of the system, the IVM molecule was subjected to a harmonic force in the direction normal to the DOPC-lipid membrane surface. This force was applied to the center of mass of the IVM structure, guiding the molecules into the DOPC membrane. This process was carried out slowly so that the two reference structures could mutually organize during the interaction process. From this classical MD trajectory, a configuration containing ivermectin within the lipid membrane was selected and considered as the initial configuration for a new round of MD simulations for thermalization. Later, a long classical trajectory was produced for statistical analysis of the properties of interest, considering the IVM–lipid membrane interaction. In the event of IVM escaping from within the lipid membrane, a new configuration was selected considering the most internal molecule in the lipid membrane. This same procedure was performed for membranes containing 115 DOPCs and 13 cholesterol molecules (DOPC+10%-CHOL), with 102 DOPCs and 26 cholesterol molecules (DOPC+20%-CHOL), and with 90 DOPCs and 38 cholesterol molecules (DOPC+30%-CHOL) to evaluate the influence of cholesterol molecules during the interaction process of the IVM within the lipid membrane.

The MD simulations were performed using the Gromacs software^15^ with DOPCs and IVM modeled by the CHARMM36 force field^16^ and water molecules modeled by the TIP3P force field.^17^ Coulomb interactions were applied using the PME algorithm,^18^ and Lennard–Jones interactions were modeled with a cutoff, both with a cutoff radius of 1.2 nm. The simulations were performed considering the NPT-semi isotropic ensemble with the application of the Parrinello–Rahman model^19^ every 0.4 ps of simulation, keeping the pressure constant at 1 atm. The temperature was kept constant (*T* = 300 K) using v-rescaling^20^ applied every 0.1 ps. The integration of Newton’s equations of motion was carried out every 0.001 ps, and the number of steps taken at each stage was 2 × 10^7^ for thermalizations and 3 × 10^7^ for the production stage. A total of 3 × 10^4^ configurations were saved for statistical analysis. The MD simulations were performed with the Lincs algorithm.^21^ Only in MD simulations where the application of harmonic force on the IVM molecule was considered (pulling technique), widely used for configuration generation, umbrella sampling.^22^ In these cases, the reference vector for harmonic force application is directed along the *z*-axis, the spring constant used in the harmonic force was 2 × 10^3^ kJ/(mol nm^2^), and the application rate was 5 × 10^–2^ nm/ps, values already tested in other works.^23,24^ Visual analysis was performed using the VMD program,^25^ while specific analyses were performed with the SuAVE program.^26^

It is important to emphasize that the present simulations were designed under the thermodynamic equilibrium, in which a single sufficiently long trajectory provides statistically reliable sampling of the system’s configurational space. The choice of including one ivermectin molecule per bilayer should be understood as a conceptual model that isolates and highlights the molecular determinants of ivermectin–lipid interactions, avoiding spurious drug–drug aggregation effects. This reductionist approach has been widely adopted in computational studies aimed at mechanistic insight and provides a controlled framework to probe how cholesterol modulates the embedding and stabilization of ivermectin within lipid membranes. Convergence was verified by energy and thermodynamics terms along the trajectory, which reached stability in the production phase (see the Supporting Information, Figures S1–S3).

### 2.3 Liposome Preparation

Liposomes were prepared using the lipid film hydration method followed by extrusion. SPC at 40 mM plus CHOL at varying molar ratios (0, 10, 20, and 30 mol %) and DOPC at 40 mM plus CHOL at 10 mol % were dissolved in chloroform in round-bottom flasks. The solvent was evaporated at 40 °C using a rotary evaporator (RV10, IKA, Germany) to form a lipid film, which was then kept under vacuum overnight to ensure complete solvent removal. The lipid film was hydrated with 10 mL of 0.9% NaCl buffer solution. Following hydration, the liposome samples were extruded through polycarbonate membranes (Whatman, UK) with a 100 nm cutoff at 55 °C under 200 Psi nitrogen pressure to obtain unilamellar liposomes with an average diameter of 90–120 nm and a polydispersity index (PdI) of <0.2. After 15 extrusion cycles, DSPE-PEG 2000 was postinserted at a concentration of 2 mM.

### 2.4 Liposome Characterization

The obtained liposomes were characterized by their average diameter, polydispersity index (PdI), and zeta potential using dynamic light scattering (DLS) and electrophoretic mobility with a Zetasizer Nano-ZS instrument (Malvern Panalytical, UK). Particle concentration was assessed by nanoparticle tracking analysis (NTA) using a NanoSight NS500 system (Malvern Panalytical, UK). Additionally, the membrane fluidity of the liposomes was evaluated by using electron spin resonance (ESR) spectroscopy. ESR measurements were performed on a CW-EPR system (EMXPlus, Bruker, Germany) operating in the X-band (approximately 9.4 GHz) with a standard resonator for a high time resolution. Spectrometer parameters included: a microwave power of 2 mW, a modulation frequency of 100 kHz, a modulation amplitude of 1 G, a magnetic field scan of 100 G, a scan time of 168 s, and a detection time constant of 41 ms. All spectra were recorded at room temperature. Membrane fluidity was assessed before and after ivermectin (IVM) capture using the 5-doxyl-stearic acid (5-DSA) spin label. A mixture of 60 μL of each liposome formulation and 0.5 μL of 5-DSA (5 mg/mL) was placed in a sealed glass capillary, which was then inserted into the resonant cavity of the ESR system. The maximum hyperfine splitting parameter (2A_||_) was used as an indicator of membrane fluidity and was obtained directly from the experimental 5-DSA spectra, as previously described.^27,28^

### 2.5 Quantitative Determination of Ivermectin

IVM quantification was performed by using high-performance liquid chromatography with diode array detection (HPLC-DAD). Analyses were conducted on an Agilent 1260 Infinity system (Agilent Technologies, USA) equipped with a Zorbax SB-C18 column (150 × 4.6 mm, 5 μm particle size). The chromatographic separation employed an isocratic mobile phase consisting of water and acetonitrile (10:90, v/v) at a flow rate of 1.5 mL/min and a column temperature of 40 °C. The injection volume was set at 20 μL, and detection was carried out at 245 nm. Analytical method validation followed the criteria established by Resolution No. 166 of 2017 of the Brazilian Health Regulatory Agency (RDC 166/17, ANVISA).

### 2.6 In Vitro Entrapment of Ivermectin into Lipid Bilayers

The evaluation of IVM capture by liposomes was conducted using two groups: one group with liposomes diluted in 0.9% NaCl solution (1:1, v/v) and the other with liposomes diluted in human plasma (1:1, v/v), both adjusted to a final liposome concentration of 20 mM. Liposomes were incubated at 25 °C in a horizontal shaker at 100 rpm with a supersaturated IVM solution (2 mg/mL) prepared in 0.9% NaCl. After 24 h of incubation, liposomes were separated from nonencapsulated IVM by centrifugation at 1500×*g* for 5 min. Aliquots (50 μL) of the supernatant were diluted with acetonitrile (950 μL), and the ivermectin concentration was determined by HPLC-DAD.

### 2.7 Statistical Analysis of Experimental Data

Results were expressed as mean ± standard deviation. Statistical analyses were performed by two-way ANOVA, followed by Tukey’s post hoc test for multiple comparisons among three or more means. All analyses were conducted using GraphPad Prism version 9.3 (GraphPad Software Inc., La Jolla, CA, USA). *p*-values less than 0.05 (*p* < 0.05) were considered significant.

## 3 Results

### 3.1 Theoretical Prediction

Simulation boxes containing predefined mixtures of DOPC and CHOL were constructed to model the behavior of the lipid phase of plasma membranes upon interaction with IVM. A representation of the DOPC–CHOL simulation systems, excluding water molecules, is shown in Figure 1. Figure 2 presents a representative configuration of the lipid–ivermectin system, including the aqueous phase, obtained from classical molecular dynamics simulations.

**Figure 1:**
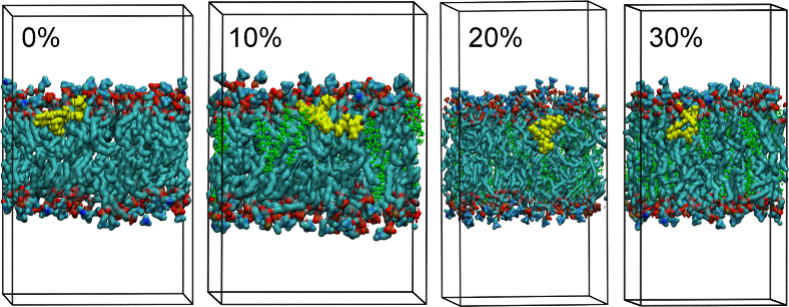
Representative configurations showing the ivermectin (IVM, yellow) molecule embedded in DOPC-lipid membranes (DOPC = red and dark green) containing increasing amounts of cholesterol (CHOL = light green): 0, 10, 20, and 30%. The 0% cholesterol system corresponds to a membrane composed purely of DOPC lipids with no cholesterol present. Water molecules fill the remaining simulation box but are omitted for the sake of clarity.

**Figure 2:**
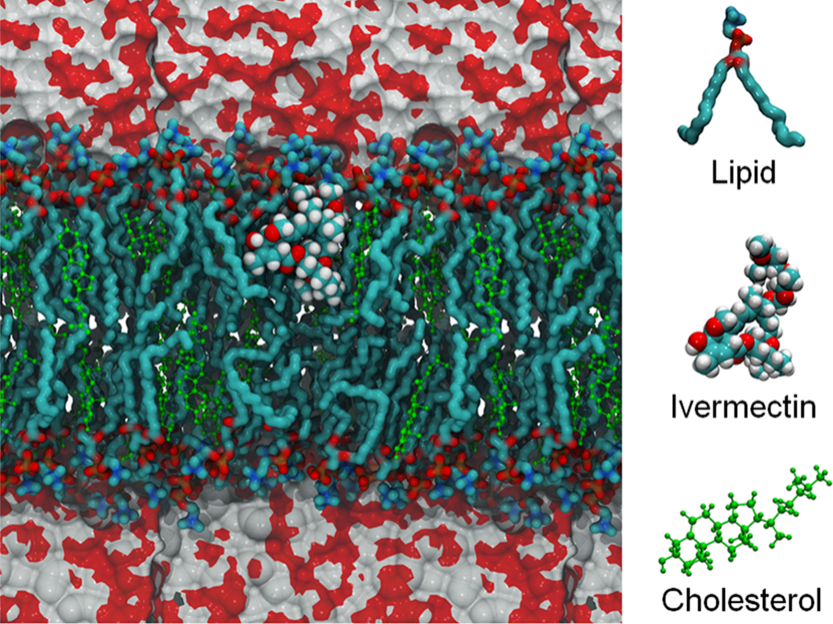
Representative configuration of the ivermectin (IVM)–DOPC system with 30% cholesterol, showing the IVM molecule positioned within the upper monolayer of the lipid membrane, just below the boundary defined by the DOPC polar headgroups and the aqueous phase (depicted by the gray-red surfaces at the top and bottom of the figure). The configuration was extracted from the molecular dynamics simulation at thermodynamic equilibrium.

The structural analyses focused on several key parameters: DOPC membrane thickness; the distance between the centers of mass of the ivermectin molecule and the lipid membrane (calculated along the vertical axis after system translation); Coulomb and van der Waals interaction energies between ivermectin and the membrane components; and the mass density profile projected along the membrane normal (*z*-axis), highlighting the position of ivermectin within the bilayer. In addition, the mean square displacement (MSD) of ivermectin was determined to assess its dispersion behavior within the system. The structure and dynamics of hydrogen bonds (HBs) formed between ivermectin and the membrane components were also analyzed to evaluate the stability of the drug–membrane interaction across the different cholesterol concentrations.

Figure 3 shows the mass density profile obtained for DOPC boxes containing 0, 10, 20, and 30% of cholesterol. By analyzing the intersection of the water and DOPC distribution curves, we can estimate the membrane thickness. For a pure DOPC system, the estimated thickness was found to be 4.16 nm, while for the DOPC-cholesterol systems, the values were 4.14 nm (CHOL-10%), 4.44 nm (CHOL-20%), and 4.60 nm (CHOL-30%). Additionally, the yellow shaded area indicates the region where the ivermectin molecule remained confined during the molecular dynamics’ simulation. For the CHOL-0% system, this region is established between 5.5 and 7.5 nm; for CHOL-10%, between 4.5 and 6.1 nm; for CHOL-20%, between 6.0 and 8.0 nm; and for CHOL-30%, between 6.1 and 8.1 nm.

**Figure 3:**
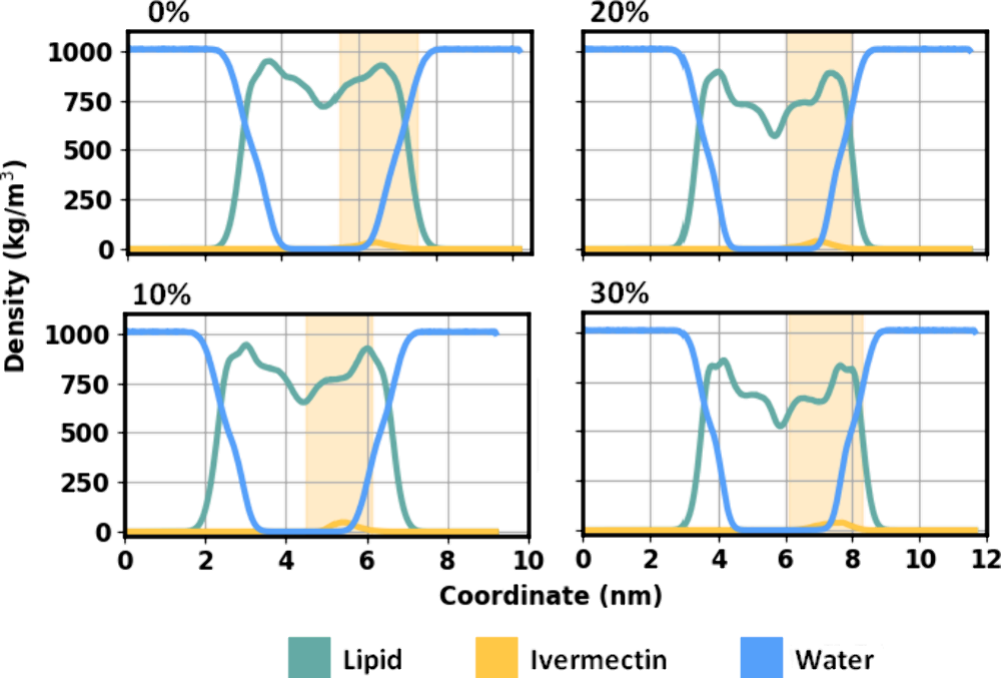
Average mass density profiles (projected along the *z*-axis of the simulation box) for the system components in DOPC bilayers containing 0, 10, 20, or 30% cholesterol. The mass density of the water molecules (blue) is zero within the region occupied by the lipid membrane (green). Membrane thickness was estimated from the distance between the intersections of the water and lipid density profiles, corresponding to the hydrated polar surfaces of the bilayer. The spatial distribution of the ivermectin molecule within the membrane is highlighted in yellow.

Membrane thickness was also analyzed using the SuAVE software, and the thicknesses obtained for each lipid system simulated were: 4.19 nm (CHOL-0%), 4.36 nm (CHOL-10%), 4.54 nm (CHOL-20%), and 4.63 nm (CHOL-30%). These results indicate that after 30 ns of simulation, the ivermectin molecule remains embedded in the interior of the DOPC membrane, with a variable distance from its center of mass to the hydrophobic core of the lipid bilayer. This can be seen in Figure 4. For pure DOPC bilayers, the average distance between IVM–membrane center of mass was found to be 1.24 nm, while for bilayers containing 10, 20, and 30% cholesterol, the IVM–membrane average distances were 1.09, 1.31, and 1.54 nm, respectively. These results suggest a tendency for ivermectin to embed more deeply within the membrane when 10% cholesterol is present.

**Figure 4:**
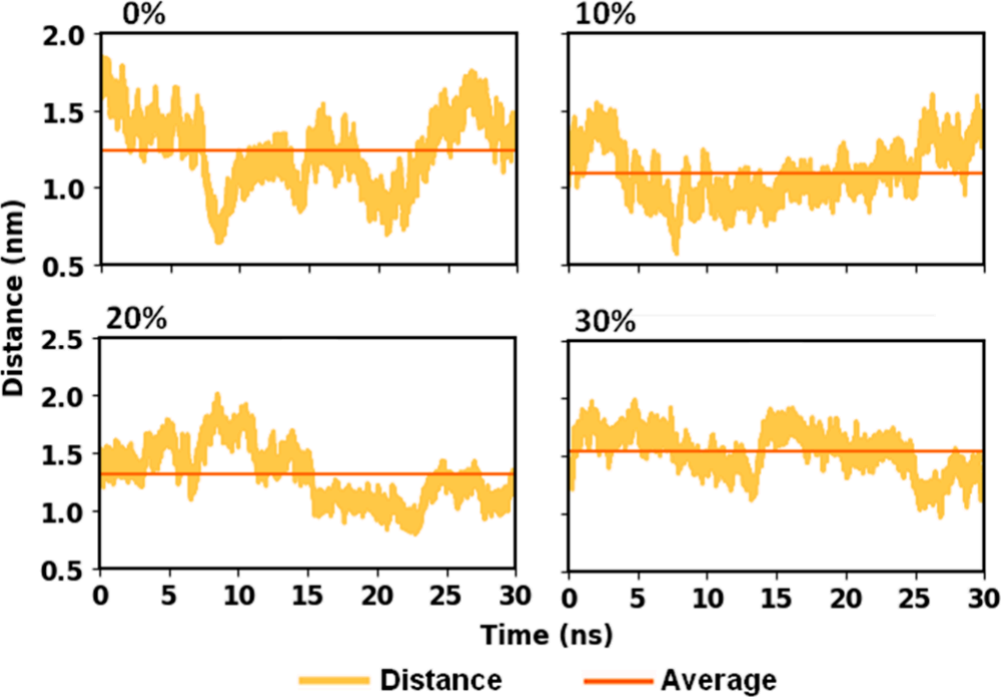
Time evolution of the distance between the centers of mass of ivermectin and the lipid membrane, measured along the vertical axis after system translation, across 30,000 configurations collected during the molecular dynamics simulation at thermodynamic equilibrium. The red line indicates the average distance over all configurations. The data correspond to systems with increasing cholesterol content (0, 10, 20, and 30%), with 0% representing the absence of cholesterol in the membrane.

The analysis of Coulomb and van der Waals energies is essential for understanding the interactions between ivermectin and the DOPC membrane, as these forces play a key role in the stabilization of molecular structures. For each simulated system, the sum of Coulomb interactions between IVM–DOPC and IVM–CHOL, as well as the sum of van der Waals interactions between IVM–DOPC and IVM–CHOL, is presented in Figure 5. For Coulomb energy, our results were −84.99, −83.18, −95.57, and −95.34 kJ/mol for the systems containing 0, 10, 20, and 30% cholesterol, respectively. The DOPC membrane containing 10% cholesterol exhibited a variation of 2.13% in relation to the cholesterol-free system, while the relative variations obtained for the 20 and 30% DOPC–CHOL boxes were 12.45 and 12.18%, respectively. Thus, based on pure Coulomb energy analysis, it can be inferred that for the membrane containing 10% of cholesterol the ivermectin molecule is positioned further from the DOPC polar head groups. On the other hand, our MD simulations revealed van der Waals energies of −294.11, −333.13, −308.52, and −291.28 kJ/mol for the systems containing 0, 10, 20, and 30% cholesterol, respectively. In comparison with the free-cholesterol system, CHOL-10% presented a variation of −13.27%, while values of −4.90 and 0.96% were found for CHOL-20% and CHOL-30% boxes, respectively. These results point to stronger hydrophobic interactions of ivermectin with DOPC molecules at 10% cholesterol, which is consistent with the observed insertion depth. While this suggests a favorable stabilization environment, the magnitude of the differences should be considered within the context of statistical uncertainty.

**Figure 5:**
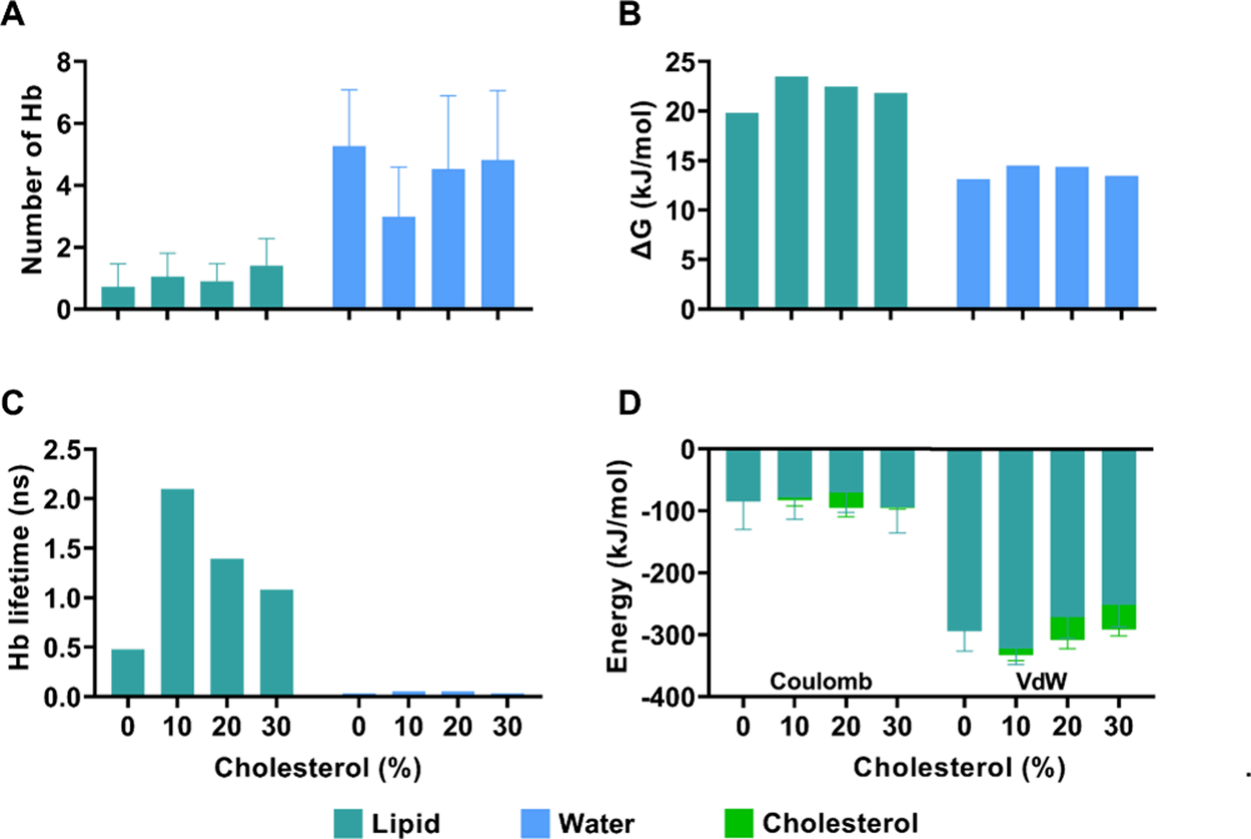
(A) Average number of hydrogen bonds formed between ivermectin (IVM) and lipids and between IVM and water molecules for each system with different cholesterol concentrations in the DOPC bilayer. (B) Energy required to break hydrogen bonds between IVM–lipid and IVM–water pairs. (C) Lifetime of hydrogen bonds formed between IVM and lipids and between IVM and water molecules. (D) Coulomb and van der Waals (vdW) interaction energies between IVM and lipids and between IVM and cholesterol molecules.

The mean square displacement (MSD) results, which reflect the mobility of the ivermectin molecule in the system, were found to be 0.0046 (±0.0018), 0.0019 (±0.0200), 0.2989 (±0.2665), and 0.1236 (±0.1440) × 10^–5^ cm^2^/s for the systems containing 0, 10, 20, and 30% cholesterol, respectively (see the Supporting Information, Figure S4, for MSD curves). The system containing 10% cholesterol showed the lowest mobility values among the tested conditions, which may indicate increased stabilization of ivermectin in this environment and thus should be interpreted as a trend.

Finally, an analysis of the structure and dynamics of hydrogen bonds (HBs) is shown in Figure 5, where one can see the behavior of the average number of HBs and their lifetimes for IVM–DOPC and IVM–water interactions. The average numbers of HBs for IVM–DOPC were 0.72 (CHOL-0%), 1.04 (CHOL-10%), 0.90 (CHOL-20%), and 1.40 (CHOL-30%). Regarding the IVM–water HBs, the results were 5.264, 2.989, 4.531, and 4.825 HBs for the systems containing 0, 10, 20, and 30% cholesterol, respectively. The HB lifetimes for IVM–DOPC were 0.48 (CHOL-0%), 2.10 (CHOL-10%), 1.39 (CHOL-20%), and 1.08 (CHOL-30%) ns, indicating that in the 10% cholesterol system the HBs formed with DOPC headgroups exhibited comparatively longer lifetimes. This tendency may contribute to a more stable interaction pattern. In a complementary manner, the HB lifetimes for IVM–water were 32.13, 55.71, 52.30, and 36.55 ps for the systems containing 0, 10, 20, and 30% cholesterol, respectively, indicating that the IVM–water interaction is less stable over time.

This set of results obtained theoretically through fully atomistic molecular dynamics simulations reveals fundamental aspects of the interactions between ivermectin and DOPC membranes with different cholesterol concentrations. This demonstrates that the inclusion of cholesterol significantly influences the structural and dynamic properties of the membrane to the extent that it either favors or hinders the anchoring of ivermectin within the membrane structure. Our MD simulations suggested that the system containing 10% cholesterol displayed characteristics that may favor ivermectin retention within the membrane. Taken together with experimental observations, this result provides a plausible mechanistic explanation. These include a higher degree of insertion into the membrane compared to models with higher cholesterol concentrations and the cholesterol-free model.

The highlighted case shows reduced mobility of ivermectin, which decreases the likelihood of the molecule escaping the membrane structure as well as higher van der Waals interaction energy (the predominant energy in ivermectin–membrane interactions), further reinforcing the stabilizing effect of hydrophobic forces at this cholesterol concentration. Additionally, the presence of hydrogen bonds between ivermectin and the DOPC molecules further confirms the enhanced stability of the 10% cholesterol system. These interactions exhibit longer lifetimes and higher bond dissociation energies, indicating the anchoring of ivermectin to the polar heads of DOPCs, with the molecule deeply embedded in the membrane.

To further evaluate the statistical robustness of the MD results, all observables were recalculated using a block-averaging approach, and the corresponding hydrogen-bond statistics and interaction energies are reported in the Supporting Information (Figures S5 and S6). The hydrogen-bond counts and lifetimes obtained from subtrajectories reproduce the same cholesterol-dependent trends observed in the main analysis, despite moderate variations in absolute values arising from finite-time sampling. In particular, the enhanced number and lifetime of IVM–DOPC hydrogen bonds at 10% cholesterol is preserved across subtrajectories, while IVM–water hydrogen bonding remains comparatively short-lived, indicating a stable anchoring of ivermectin at the lipid–water interface. Consistently, the block-averaged interaction energies show the same relative balance between coulombic and van der Waals contributions, confirming that the predominance of hydrophobic stabilization at intermediate cholesterol content is not sensitive to temporal partitioning of the trajectory. These results corroborate that the trends presented above are preserved when the trajectory is treated as a statistically independent block, reinforcing the reproducibility and physical consistency of the simulations.

### 3.2 Experimental Verification

Liposomes were characterized based on key physicochemical parameters, including mean size, PdI, zeta potential, and particle concentration, with the results summarized in Table 1. The formulations consistently demonstrated a mean diameter of approximately 110 nm, a PdI value below 0.1, a near-neutral zeta potential, and a particle concentration of approximately 10^14^ particles/mL. These characteristics are indicative of well-formed vesicles with a narrow size distribution, favorable surface charge, and high particle concentration, all of which are essential for the efficient delivery and stability of therapeutic liposomal formulations, as reported in the literature.^29^

**Table 1:** Characterization of the Liposomal Formulations^a^

	SPC				DOPC
	cholesterol				
parameters	0%	10%	20%	30%	10%
**mean size (nm)**	103 ± 5	107 ± 2	105 ± 2	115 ± 4	92 ± 4
**PdI**	0.05 ± 0.01	0.08 ± 0.01	0.07 ± 0.01	0.10 ± 0.01	0.09 ± 0.01
**zeta potential (mV)**	–4.0 ± 0.3	–5.1 ± 0.1	–5.0 ± 0.4	–4.4 ± 0.3	–1.5 ± 0.2
**concentration** **(10** ^**14**^ **particles/mL)**	2.00 ± 0.05	1.79 ± 0.05	2.77 ± 0.06	1.59 ± 0.08	1.15 ± 0.78

a Mean size, polydispersity index (PdI), zeta potential, and particle concentration are reported. Data are presented as the mean ± standard deviation (SD) (*n* = 3).

To compare experimental findings with MD simulations, SPC liposomes were exposed to IVM, and the amount of IVM captured by each formulation was quantified. As shown in Figure 6, liposomes containing 10% cholesterol exhibited the highest IVM capture efficiency. However, a progressive decrease in the capture capacity was observed as the cholesterol content in the bilayer increased beyond this concentration. A similar trend was noted when the experiments were conducted in the presence of plasma.

**Figure 6:**
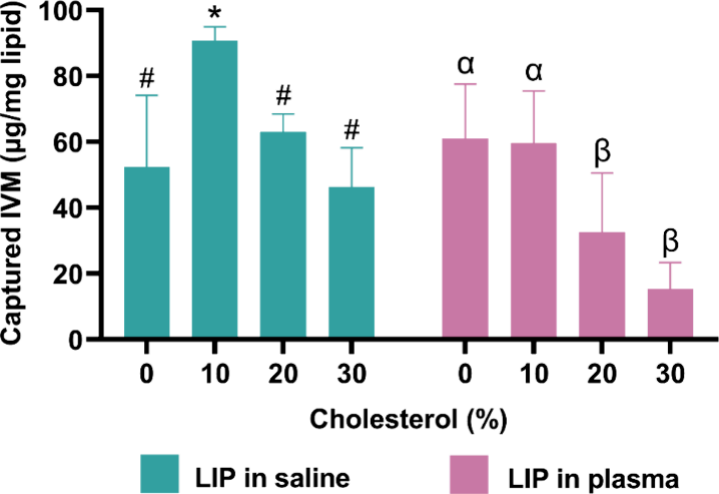
Ivermectin (IVM) captured by liposomes (LIP) formulated with different cholesterol contents. Liposomes (20 mM) were incubated in saturated IVM solutions prepared in either 0.9% NaCl (saline) or 50% plasma. Data are presented as mean ± standard deviation (SD) (*n* = 6). Distinct symbols indicate statistical differences within each medium (one-way ANOVA with Tukey’s post hoc test, *p* < 0.05).

The molecular dynamics of the lipid bilayers was further investigated by evaluating the maximum hyperfine splitting distance (2A_||_) of 5-DSA spin labels incorporated into the membranes (Figure 7). As expected, increasing cholesterol concentrations led to a significant rise in 2A_||_ values, indicating a reduced molecular motion and increased membrane rigidity due to enhanced lipid packing. Notably, liposomes exposed to saturated IVM solutions displayed an additional increase in their 2A_||_ values. This complementary stiffening effect suggests that IVM incorporation into the hydrophobic core of the bilayers further restricts lipid mobility, corroborating predictions from our MD simulations.

**Figure 7:**
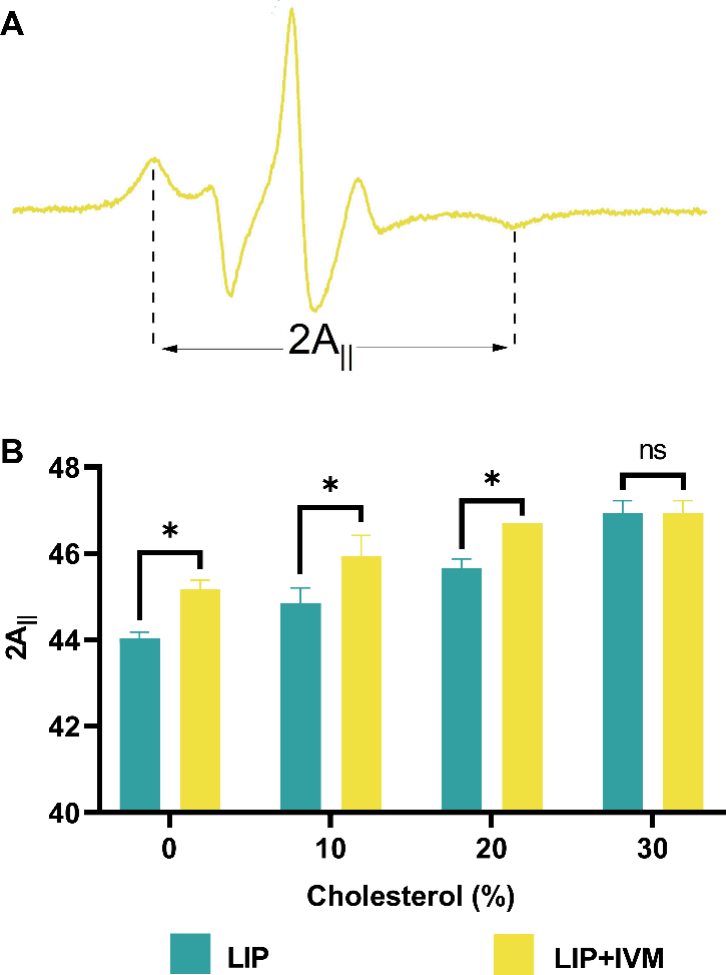
ESR spectra of spin-labeled liposomal membranes before (green) and after (yellow) ivermectin (IVM) capture. Panel (A) illustrates the outer hyperfine splitting of 5-DSA spin labels, used to calculate the 2A_||_ values. Panel (B) presents the 2A_||_ values extracted from each corresponding spectrum. Spectral intensities are normalized on the *y*-axis, and the total magnetic field range is 100 G.

To further assess the consistency between the experimental lipid system and the membrane model employed in the molecular dynamics simulations, additional experiments were performed comparing ivermectin capture by DOPC and SPC liposomes containing 10% cholesterol. As shown in Figure 8, both lipid systems exhibited statistically indistinguishable ivermectin capture efficiencies under identical conditions, indicating that the presence of a heterogeneous lipid composition in SPC does not significantly alter ivermectin uptake at intermediate cholesterol content. In parallel, ESR spectroscopy was used to evaluate membrane rigidity through the 2A_∥_ parameter. Comparable 2A_∥_ values were obtained for CHOL-10% DOPC and SPC liposomes in the absence of ivermectin, suggesting a similar baseline membrane organization. Upon exposure to ivermectin, both systems displayed an increase in 2A_∥_, with DOPC liposomes showing slightly higher values, consistent with a more pronounced ordering effect in the chemically homogeneous bilayer. Importantly, these differences in membrane ordering do not translate into measurable differences in ivermectin capture efficiency, supporting the use of SPC as an experimentally relevant lipid system and DOPC as an appropriate model membrane for mechanistic interpretation.

**Figure 8:**
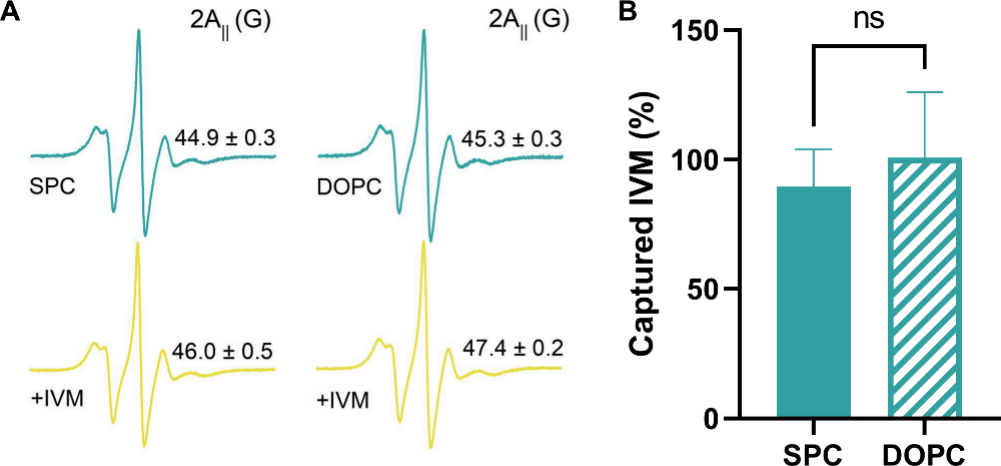
(A) ESR spectra of spin-labeled DOPC and Soy PC (SPC) liposomes containing 10% cholesterol before (green) and after (yellow) ivermectin (IVM) capture with the corresponding 2A∥ values indicated for each condition. (B) Percentage of ivermectin captured by DOPC and SPC liposomes containing 10% cholesterol. Data are presented as mean ± standard deviation (SD) (*n* = 6).

## 4 Discussion

The integrated molecular dynamics and experimental analyses consistently highlighted that membranes containing 10 mol % cholesterol provided a more favorable environment for ivermectin capture and stabilization. At this composition, the simulations showed deeper insertion of the drug, stronger van der Waals interactions, reduced mobility, and longer HB lifetimes with lipid headgroups, while experimental assays confirmed the highest association of ivermectin with liposomes. This convergence of computational and experimental observations supports the view that moderate cholesterol enrichment promotes a unique balance of membrane fluidity and packing that optimizes ivermectin retention. The capacity of lipid membranes to accommodate and stabilize hydrophobic molecules is a fundamental property with broad implications in drug delivery and molecular sequestration. While liposomal formulations are predominantly explored for controlled delivery, their ability to selectively incorporate lipophilic compounds also positions them as promising systems for drug capture and modulation of bioavailability. This study focused on ivermectin, a highly lipophilic molecule with clinical relevance in both human and veterinary medicine, whose physicochemical properties challenge conventional handling, particularly in scenarios of altered systemic distribution.

The plasma membranes of eukaryotic cells are composed of a diverse lipid repertoire, predominantly phospholipids and cholesterol.^30^ To address this complexity in a controlled manner, DOPC was selected as the structural lipid for MD simulations due to its well-defined, unsaturated phospholipid profile, facilitating the precise interpretation of lipid dynamics. In parallel, SPC-based liposomes were employed for experimental validation, providing a heterogeneous phosphatidylcholine mixture that better reflects biological membranes.^31^ Despite its compositional simplicity, DOPC exhibits membrane behavior comparable to SPC at physiological temperatures, justifying its use as a computational model.^32^ Importantly, cholesterol’s modulatory effects on bilayer properties are expected to exert similar influences across these lipid systems, supporting the extrapolation of simulation findings to SPC-based experimental models.^33^ The agreement observed between the DOPC simulations and SPC experimental data further reinforces the validity of this combined approach.

Our approach, which combines atomistic molecular dynamics with experimental validation in SPC-based liposomes, clearly demonstrates the exploratory and integrative nature of this work. The simulations provide molecular-level insight into ivermectin–cholesterol interactions, while the experiments demonstrate that these trends are reflected in real lipid vesicles. Together, these complementary methods generate solid hypotheses about how cholesterol modulates ivermectin partitioning, offering a mechanistic basis for the rational design of liposomal systems tailored for drug capture. MD simulations suggested that liposomes containing 10 mol % cholesterol may present a balance between membrane fluidity and molecular packing that favors somewhat deeper embedding and stabilization of ivermectin within the bilayer. These effects appear as trends across the MD simulations and should be interpreted in the context of the exploratory nature of the model, paving the way toward a wide range of applications. This environment favors strong van der Waals interactions and reduces the ivermectin mobility, as evidenced by diminished mean square displacement values. Concurrently, the system exhibited an increased hydrogen-bond lifetime between ivermectin and lipid headgroups, accompanied by a reduction in ivermectin–water interactions, indicating a more stable drug–membrane association.^13,34,35^

These findings align with the established role of cholesterol in condensing lipid bilayers, enhancing molecular packing, and modulating membrane dynamics.^13,14^ Moderate cholesterol enrichment promotes bilayer organization while maintaining sufficient fluidity to permit small molecule insertion, a balance that appears to be critical for optimizing ivermectin retention. Notably, higher cholesterol concentrations (20–30%) induced increased bilayer thickness and rigidity, restricting ivermectin penetration and lateral mobility, consistent with cholesterol’s condensing effects on unsaturated phospholipid membranes.^14,32^

Experimental ESR spectroscopy corroborated these observations, demonstrating a cholesterol-dependent increase in the membrane order, further accentuated by ivermectin incorporation. The maximum hyperfine splitting distance (2A_||_) values, derived from spin-labeled lipid probes, confirmed progressive membrane rigidification with increasing cholesterol content. Importantly, ivermectin itself contributed to membrane stiffening through intercalation between lipid molecules, restricting lateral lipid mobility and altering bilayer organization. The strong concordance between atomistic MD simulations and experimental observations reinforces the robustness of the findings, highlighting how both approaches converge to a consistent mechanistic picture of cholesterol-dependent ivermectin capture.

Quantitative ivermectin capture assays further validated the computational predictions, revealing that liposomes containing 10 mol % cholesterol achieved the highest drug association. Notably, these trends persisted, even in plasma-containing environments, reinforcing the robustness of the observed interactions. The presence of plasma, despite its high protein content and ivermectin’s well-documented high protein-binding affinity, did not significantly alter the liposomes’ capacity to capture ivermectin. This observation can be attributed to the experimental design, in which ivermectin was present at oversaturation concentrations. Under such conditions, a dynamic equilibrium is established between protein-bound and free ivermectin molecules. The high drug concentration ensures a continuous pool of unbound ivermectin available for partitioning into the liposomal bilayer, effectively allowing the liposomes to sequester free drug molecules despite the competitive binding environment. The use of these elevated concentrations was intentional, aimed at validating the formulation’s capacity for drug capture under challenging yet controlled conditions. This approach provided a stringent test of the liposomal system’s efficiency, thereby supporting its potential application in scenarios where efficient sequestration of excess ivermectin is desired. Previous studies have similarly demonstrated that cholesterol enrichment increases membrane order and modulates the partitioning of hydrophobic molecules, with excessive ordering impairing small molecule insertion.^34^

Despite ivermectin’s large molecular size, its high membrane–water partition coefficient (∼2700 L/kg lipids) and markedly low aqueous solubility confer a strong preference for lipid environments.^35^ These physicochemical characteristics likely underpin its efficient capture by lipid membranes, as observed in both MD simulations and experimental assays. Additionally, ivermectin’s ability to alter membrane properties has been reported in parasitic and mammalian systems, including increased plasma membrane rigidity and enhanced antiparasitic activity at lower concentrations.^27^

Collectively, these findings provide a robust foundation for the rational design of liposomal systems optimized for ivermectin capture. While the primary focus of liposomal platforms has historically centered on drug delivery, their potential as molecular scavengers for lipophilic compounds offers alternative applications, particularly in scenarios requiring the modulation of systemic drug levels. Although this study does not investigate in vivo detoxification, the mechanistic insights presented here lay the groundwork for future explorations of liposome-based sequestration strategies.

Given ivermectin’s widespread use and the clinical relevance of managing its systemic exposure in specific contexts, optimizing liposomal systems for efficient drug capture holds significant potential. The modulation of membrane properties through cholesterol content emerges as a critical parameter in fine-tuning such formulations. Future studies may expand upon these findings by incorporating more complex lipid compositions, including sphingolipids and glycosphingolipids, which modulate membrane microdomains and drug partitioning behavior. Additionally, assessing these systems under dynamic physiological conditions, such as mechanical stress or asymmetric bilayers, would further enhance the translational relevance of this approach.

## 5 Conclusions

This work elucidates the influence of the cholesterol content on the capacity of liposomal membranes to capture and retain ivermectin, integrating molecular dynamics simulations with experimental data. The results highlight that moderate cholesterol enrichment (10 mol %) optimizes bilayer characteristics, promoting ivermectin embedding and stabilization through enhanced hydrophobic interactions and membrane structuring. These findings offer a valuable foundation for the development of liposomal systems designed to modulate ivermectin partitioning in biological environments, potentially informing future applications in which effective sequestration of the drug is desired. Importantly, the strong agreement between atomistic simulations and experimental validation reinforces the robustness of these conclusions, providing a coherent and mutually supportive picture of cholesterol-dependent drug capture. Further studies are warranted to explore the translational potential of these systems in diverse biomedical contexts.

## Supplementary Material



## Notes

Molecular dynamics supplementary data, including convergence analysis, mean square displacement (MSD) profiles, and block-averaged analyses of mass density distributions, hydrogen-bond statistics, and interaction energies (PDF)
