# Supplementary material for: Cholesterol-Driven Optimization of Liposomal Systems for Ivermectin Capture: Insights from Experimental and Molecular Dynamics Studies
Source: ACS Appl Mater Interfaces. 2026 Feb 16;18(7):10832–41. doi: 10.1021/acsami.5c21365 (PMC12954670; doi:10.1021/acsami.5c21365)
Supplement: Supplementary file 1 [file am5c21365_si_001.pdf]

SUPPORTING INFORMATION

Cholesterol-Driven Optimization of Liposomal Systems for Ivermectin Capture: Insights from Experimental and Molecular Dynamics Studies

Alexandre C. M. Barros[a,#], Jader Pires[b,c,#], Karinna Mendanha[d], Lucas R. de Sousa[b,d], Bianca B. Fontanezi[b,c], Guilherme Colherinhas[d], Ana F. M. Botelho[a], Sebastião A. Mendanha[b,d], Eliana M. Lima[b,c,*]

[a] *Laboratory of Veterinary Toxicology – School of Veterinary and Animal Sciences, Federal University of Goiás, Goiânia, Goiás, 74690-900, Brazil.*
[b] *FarmaTec - Laboratory for RD&I in Pharmaceutical Nanotechnology and Drug Delivery Systems, Samambaia Technology Park, UFG, Goiânia, Goiás, 74690-631 - Brazil*
[c] *School of Pharmacy, Federal University of Goiás, Goiânia, 74690-631, Brazil.*
[d] *Institute of Physics, Federal University of Goiás, Goiânia, Goiás, 74690-900, Brazil.*
** Corresponding author: Eliana Martins Lima - emlima@ufg.br*
[#] A.C.M.B. and J.P. contributed equally to this work

Abstract: This study investigates the interactions between ivermectin (IVM) and lipid membranes with varying cholesterol contents using a combined molecular dynamic (MD) and experimental approach. DOPC bilayers containing 0%, 10%, 20%, or 30% cholesterol were simulated, and SPC liposomes were employed for experimental validation. Mass density profiles indicated that the membrane thickness increased from 4.16 nm (0% cholesterol) to 4.60 nm (30% cholesterol), while ivermectin was most deeply embedded in membranes with 10% cholesterol, with an average distance of 1.09 nm from the bilayer center. Van der Waals interaction energies were most favorable at 10% cholesterol (-333.13 kJ/mol), correlating with increased hydrogen bond lifetime (2.10 ns) between IVM and lipid molecules. Mean square displacement (MSD) analysis revealed that ivermectin exhibited the lowest mobility (0.0019×10^{-5} cm^2/s) in membranes with 10% cholesterol. ESR spectroscopy of 5-DSA-labeled SPC liposomes demonstrated a progressive increase in $2A_{||}$ values with rising cholesterol content, with additional increases following IVM incorporation. IVM capture experiments showed that liposomes containing 10% cholesterol achieved the highest drug association, consistent across saline and plasma environments. These findings provide a mechanistic basis for the rational design of liposomal systems with high ivermectin-binding capacity, with potential implications for future applications requiring the sequestration of this compound in biological environments.

Keywords: lipid membrane dynamics; drug-membrane affinity; nanocarrier design; computational modeling; sterol content variation.

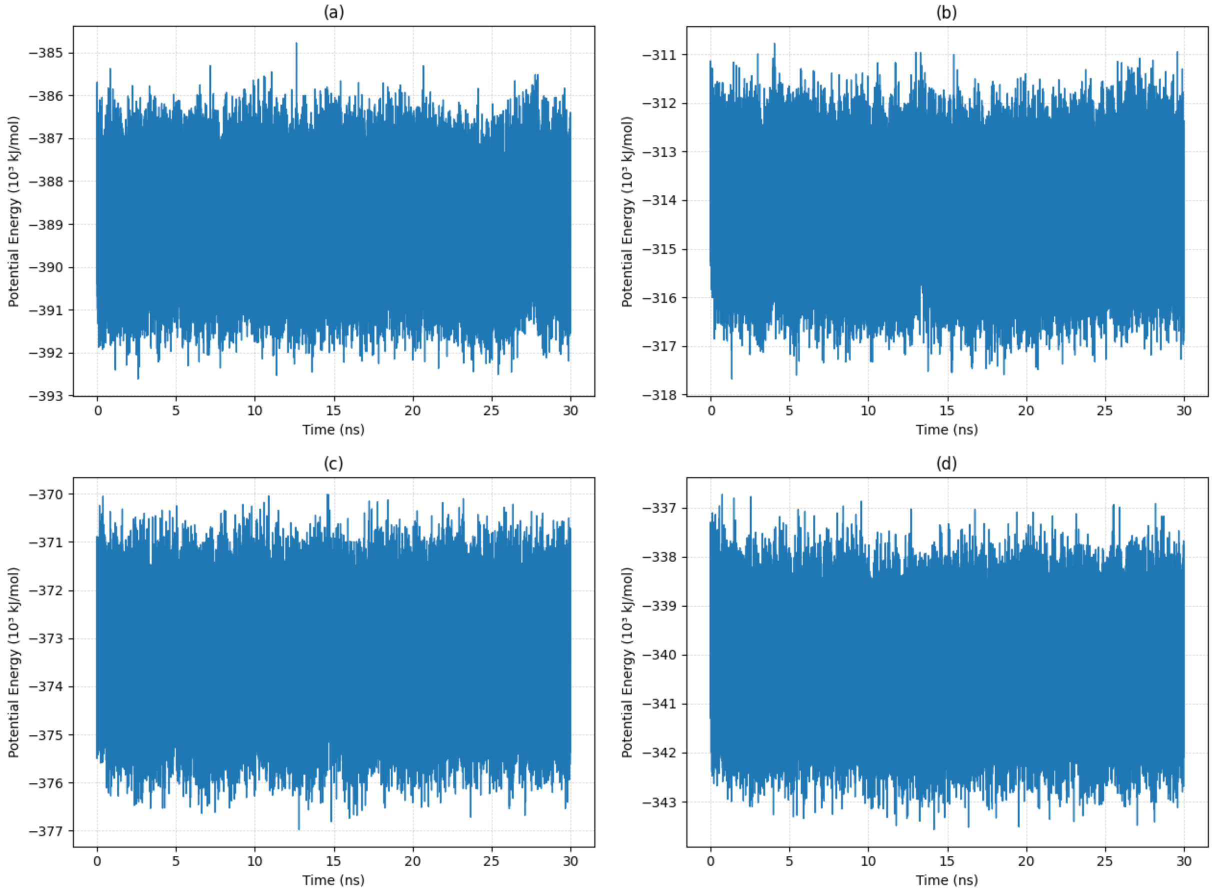

Figure S1: *Potential Energy (10^3kJ/mol) for all system simulated. Results obtained in the production phase for 10^4 configurations. (a) Pure DOPC; (b) DOPC-10% cholesterol; (c) DOPC-20% cholesterol; (d) DOPC-30% cholesterol.*

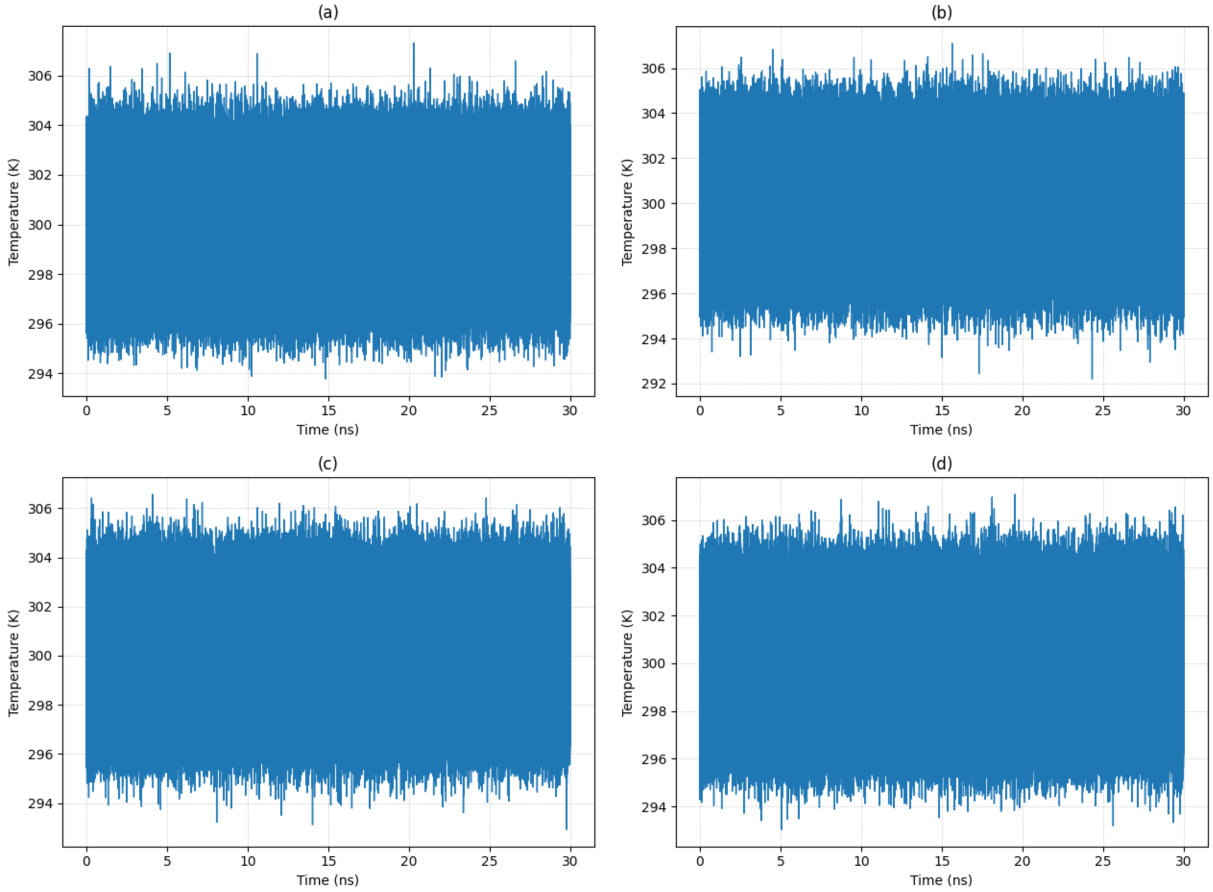

Figure S2: *Temperature (K) for all system simulated. Results obtained in the production phase for 10^4 configurations. (a) Pure DOPC; (b) DOPC-10% cholesterol; (c) DOPC-20% cholesterol; (d) DOPC-30% cholesterol.*

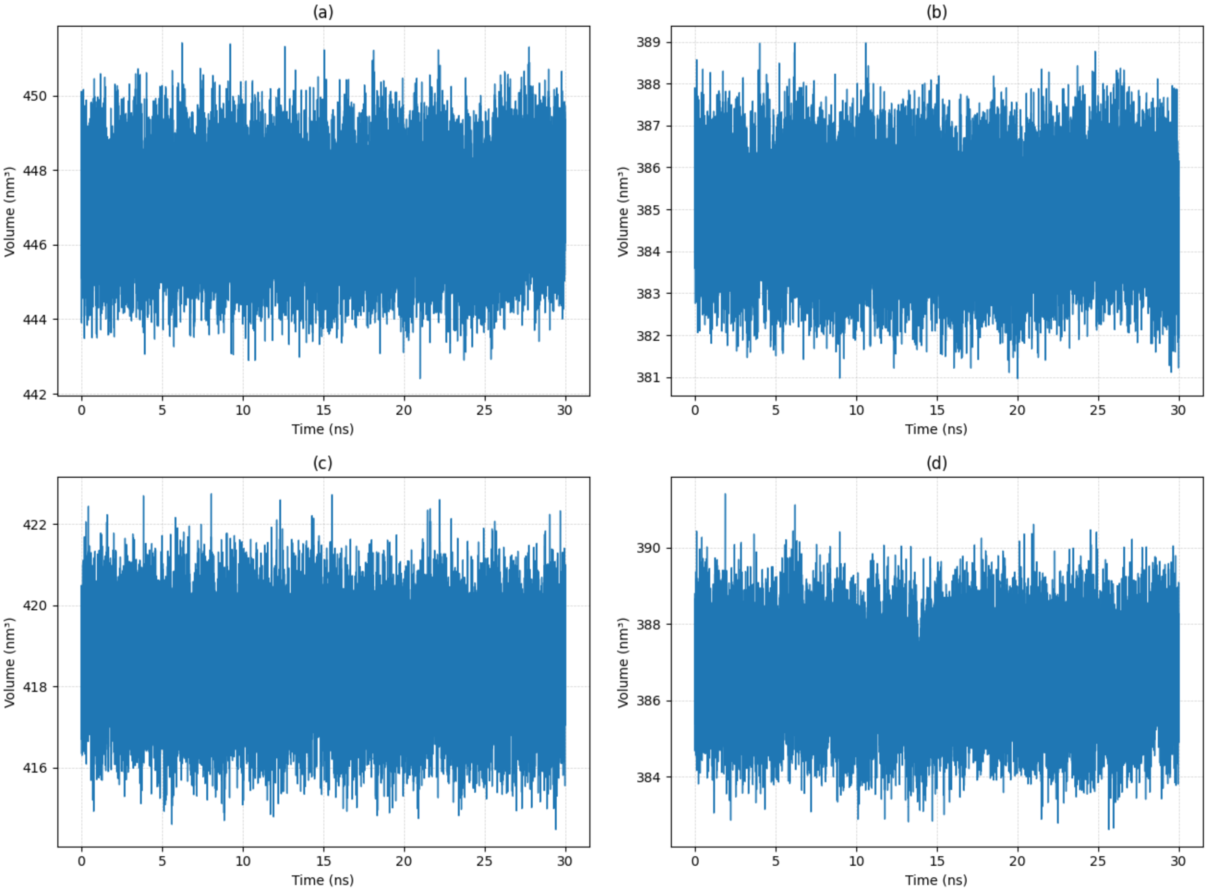

Figure S3: *Volume of system box (nm³) for all system simulated. Results obtained in the production phase for 10^4 configurations. (a) Pure DOPC; (b) DOPC-10% cholesterol; (c) DOPC-20% cholesterol; (d) DOPC-30% cholesterol.*

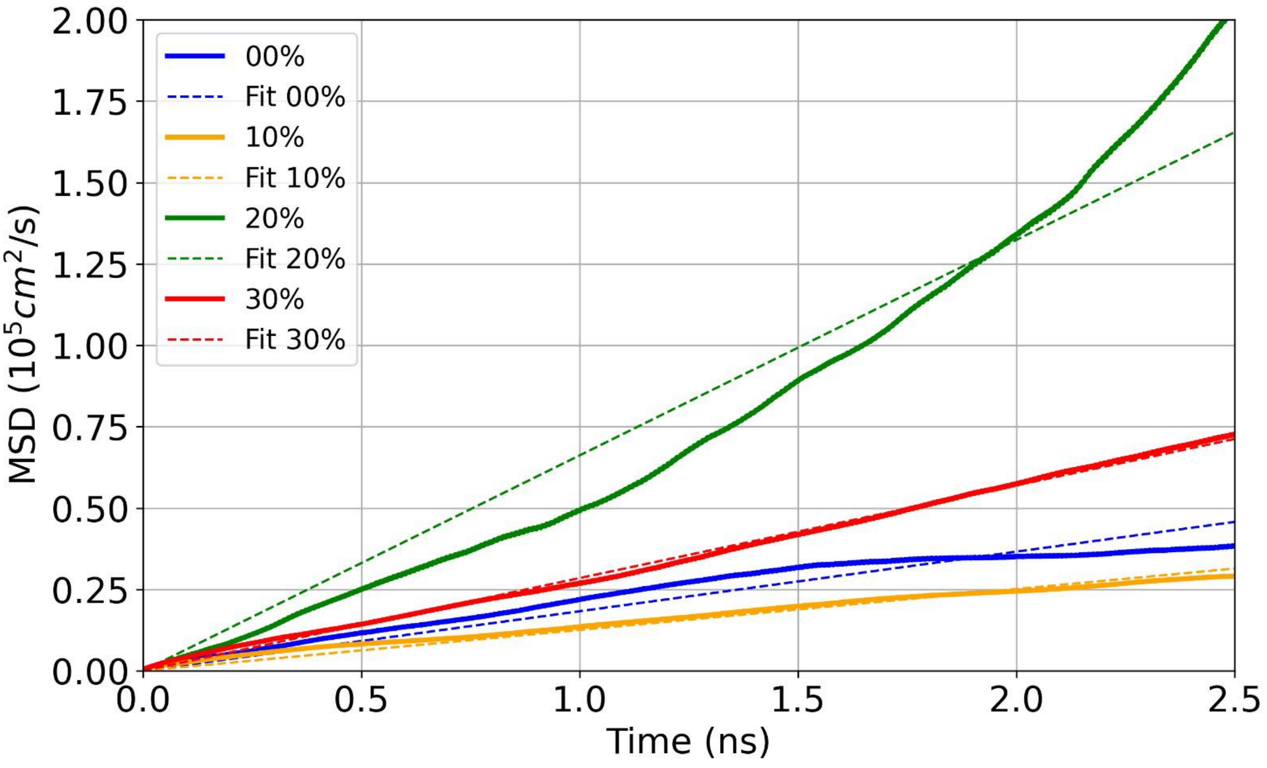

Figure S4: *Mean squared displacement (MSD) of ivermectin obtained from molecular dynamics simulations in pure DOPC bilayers (0%) and in DOPC bilayers containing 10%, 20%, and 30% cholesterol. The dotted lines indicate the linear fits used to extract the diffusion coefficients, reflecting the relative mobility of ivermectin in each system.*

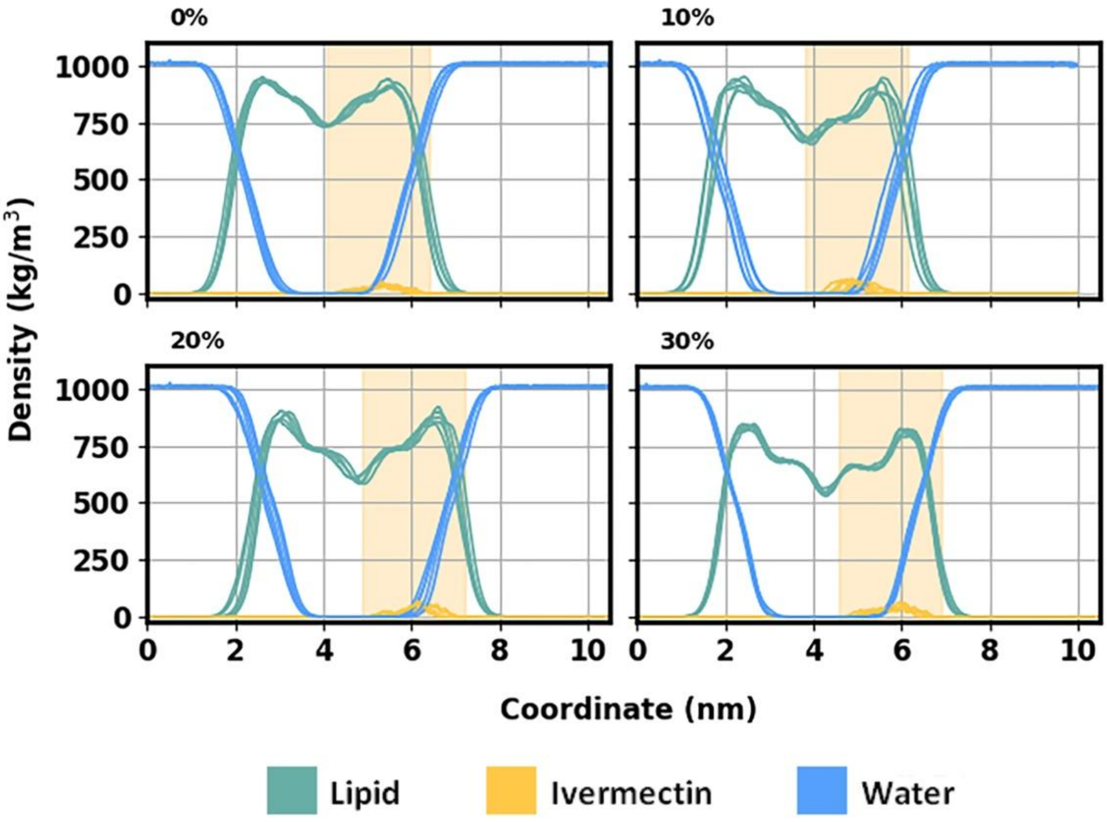

Figure S5: *Mass density profiles (projected along the Z-axis of the simulation box) for the system components in DOPC bilayers containing 0%, 10%, 20%, or 30% cholesterol obtained from the sub-trajectory analysis. The mass density of water molecules (blue) is zero within the region occupied by the lipid membrane (green). Membrane thickness was estimated from the distance between the intersections of the water and lipid density profiles, corresponding to the hydrated polar surfaces of the bilayer. The spatial distribution of the ivermectin molecule within the membrane is highlighted in yellow.*

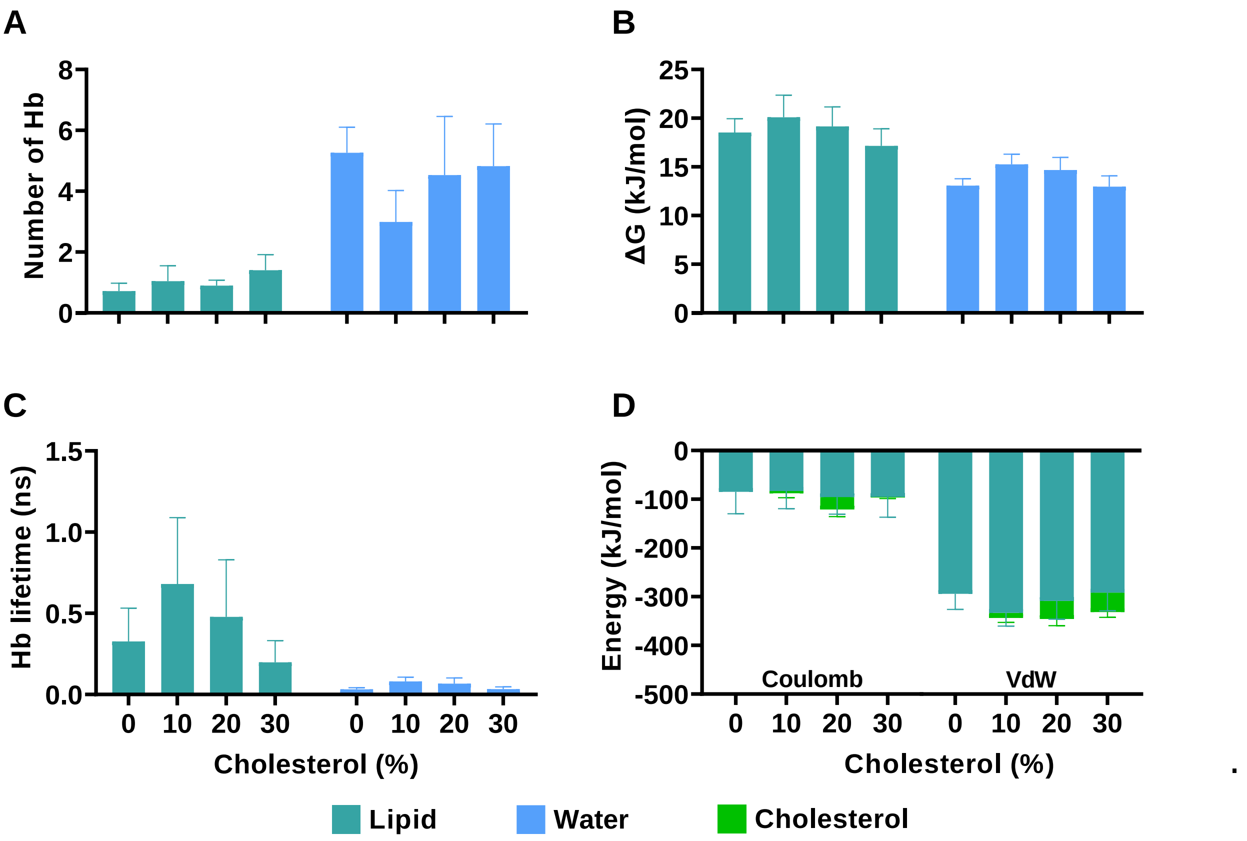

Figure S6: *(A) Average number of hydrogen bonds formed between ivermectin (IVM) and lipid molecules, and between IVM and water, as a function of cholesterol concentration in DOPC bilayers. (B) Block-averaged hydrogen-bond dissociation energy for IVM–lipid and IVM–water interactions obtained from sub-trajectory analysis. (C) Block-averaged hydrogen-bond lifetimes for IVM–lipid and IVM–water pairs derived from sub-trajectory analysis. (D) Coulombic and van der Waals (vdW) interaction energies between IVM and lipids and between IVM and cholesterol molecules.*
